# AI-augmented prenatal care: a dual-modal fetal health assessment system integrating cardiotocography and uterine contraction synergy

**DOI:** 10.3389/fphys.2025.1638788

**Published:** 2025-09-29

**Authors:** Tianxin Qiu, Xinghe Zhou, Jun Zhou, Chunxia Lin, Shiling Jiang, Hui Cheng, Xinhao Wang, Qingshan You

**Affiliations:** ^1^ Department of Obstetrics, West China Longquan Hospital Sichuan University, The First People’s Hospital of Longquanyi District Chengdu, Chengdu, Sichuan, China; ^2^ Faculty of Science, Civil Aviation Flight University of China, Chengdu, Sichuan, China

**Keywords:** fetal heart rate monitoring, artificial intelligence, bimodal analysis, DenseNet121-SK, AI-assisted decision-making

## Abstract

**Introduction:**

Fetal heart monitoring (FHR) is a critical tool for assessing fetal health, but traditional methods rely on subjective physician interpretation, exhibiting significant variability that can lead to misdiagnosis and overtreatment. Artificial intelligence (AI) technology offers a novel approach to address this issue, yet existing research predominantly utilizes unimodal (FHR-only) data, failing to align with clinical guidelines emphasizing “bimodality analysis of fetal heart rate and uterine contractions (UC).” This study aims to develop a deep learning-based bimodal intelligent monitoring system to enhance the accuracy and clinical utility of fetal health assessment.

**Methods:**

The research team constructed the first fetal heart-contraction bimodal clinical dataset for Chinese pregnant women (n = 326). Based on the DenseNet121 architecture, a selective attention mechanism (SK module) was introduced, proposing the DenseNet121-SK model. Standardized FHR and UC signals were extracted using image processing techniques. Dense connections and the SK module dynamically fused multi-scale features (e.g., transient fluctuations and contraction cycle associations). The model employed lightweight design during training to enhance physician usability.

**Results:**

(1) Dual-modality input significantly outperformed single-modality input, achieving a classification AUC of 0.944 (vs. 0.812 for single-modality), validating the clinical value of multi-parameter collaborative interpretation; (2) The SK module simulated obstetricians' multi-scale cognition, achieving 95.88% accuracy with 100% recall for abnormal cases; (3) The system effectively reduced subjective interpretation variability, providing technical support for minimizing overtreatment.

**Discussion:**

This study achieves a balance between clinical interpretability and high performance through lightweight AI design (only 8.3 million parameters) and dual-modality data fusion, making it particularly suitable for resource-constrained primary care settings. Future work should further optimize generalization capabilities through multicenter validation and explore integration with large language models to generate standardized reports. These findings provide important references for optimizing perinatal healthcare resources and AI-assisted decision-making.

## 1 Introduction

FHR monitoring is a widely used method to assess the condition of the fetus during pregnancy, labor and delivery. In high-income countries, continuous fetal heart rate monitoring with a fetal heart monitor (CTGs) is commonly performed for deliveries classified as high-risk. In contrast, in low-income and lower-middle-income countries (LMICs), intermittent measurements are the usual method for all deliveries. Intermittent measurements are usually performed using a Pinard stethoscope or a hand-held Doppler device. Guidelines recommend (Lewis et al., 2015) that auscultation of the fetal heart rate should be performed every 15–30 min during the first stage of labor, and every 5–15 min during the second stage of labor, and each auscultation should also last at least 1 min. However, due to the complexity of fetal physiologic dynamics (Costa Santos et al., 2005; Hruban et al., 2015), common standards for visual interpretation of fetal heart rate signals can lead to significant subjective variability. To minimize diagnostic errors, obstetricians perform multiple subjective assessments. As a result, the incidence of untimely cesarean sections (CS) is increasing, largely due to subjective errors (Steer, 2008). This is the main significance of designing an automatic analysis of fetal heart rate signals in this study.

In recent years, with the rapid development of machine learning and deep learning, artificial intelligence (AI)-based fetal heart rate monitoring and analyzing systems have provided new ideas to address untimely cesarean deliveries caused by subjective interpretation bias in traditional monitoring. Traditional fetal heart monitoring relies on physicians’ experience in interpreting fetal heart rate curves (e.g., baseline variability, deceleration type, etc.), but the consistency of interpretation among different physicians is not high due to individual differences and visual fatigue, and it is prone to triggering over-intervention (Madiraju et al., 2025). In machine learning approaches, a process of signal processing, feature extraction, salient feature selection, training, and final classification of the model is usually used. Complex manually introduced features are used in these methods. For example, Czabanski et al. (2012) used weighted fuzzy scoring (WFS) combined with support vector (SVM) to predict neonatal acidosis and obtained 92% accuracy and 88% quality index. O’sullivan et al. (2021) proposed a method for detecting fetal distress based on autoregressive sliding average (ARMA) modeling and machine learning, achieving a 0.86 AUC. Fanelli et al. (2013) introduced a phase-corrected signal averaging nonlinearity parameter for the quantitative assessment of fetal anomalies and achieved an AUC of 75%. Cömert et al. (2018) applied a neural network and obtained an accuracy of 92.40%, a sensitivity of 95.89% and a specificity of 74.75%, as well as the method recently proposed by Karmakar et al. (2025) recently proposed an automated classification model for fetal health status by integrating machine learning algorithms such as gradient boosting classifiers and random forests obtained 93.41% accuracy.

In contrast to traditional machine learning methods, more research is currently being conducted based on Convolutional Neural Networks (CNNs) and Long Short-Term Memory Networks (LSTMs) in deep learning. Since fetal heartbeat maps are time-series data, but often presented as two-dimensional images (time on the horizontal axis, fetal heart rate and contractions on the vertical axis), using CNNs to automatically extract spatio-temporal features (e.g., local fluctuations, cyclic patterns) through multilayered convolutional kernels and relying on the sliding-window mechanism to capture local temporal dependencies can dramatically improve the recognition accuracy of fetal heartbeat maps. Due to these advantages, CNNs have been used to design various screening and assistive tools, e.g., Li et al. (2018) proposed 1D-CNN and obtained 93.24% accuracy to classify FHR signals. Liu M. et al. (2021) designed a hybrid CNN-BiLSTM network based on the attention mechanism. Lin et al. (2024) developed the first automated long term prenatal FHR analysis system LARA, which is based on deep learning analysis system LARA, which generates risk distribution maps (RDM) and overall risk index (RI) through 1D-CNN model combined with sliding-window information fusion technique, which has an AUC of 0.872 on the test set.

Although the above methods through machine learning or deep learning have achieved more or less good results, researchers are not uniform in the standard of the data, for example, some people artificially introduce features to let the model learn, or use a one-dimensional array of fetal heart rate as the input of the model to learn, but usually doctors use the intuitive graph of the change curves of the fetal heart rate and the contraction rate to interpret. Therefore, in this study, in order to minimize the criteria for distinguishing normal and abnormal fetal heart rate, we innovatively use images as the dataset, which contain two curves of fetal heart rate and contraction rate, in order to be closer to the needs of clinical practice.

## 2 CTG interpretation standard

This chapter systematically describes the core interpretation criteria of CTG, which is divided into three parts: firstly, it clarifies the terms and definitions of CTG (section 2.1), which lays the foundation for the subsequent analysis; secondly, it explains in detail the categorization and interpretation of the CTG graphs during labor (section 2.2), including the characteristics of the typical waveforms and their clinical significance; and finally, it discusses the key role of UC in the fetal heart rate (section 2.3), and analyzes the potential mechanism of its impact on the changes of the fetal heart rate rate changes.

### 2.1 CTG terms and definitions

Baseline: the average fetal heart rate that fluctuates within 5 beats/min in 10 min, excluding acceleration, deceleration and significant variability; the normal FHR baseline range is 110–160 beats/min; the baseline must be a graph that lasts for more than 2 min in any 10 min, and the graph can be discontinuous; if the baseline is uncertain during the observation stage, the baseline can be determined by referring to the graph of the previous 10 min; of which (1) fetal tachycardia (tachycardia): refers to the fetal heart baseline >160 beats/min lasting ≥10 min. If the baseline is uncertain during the observation phase, the baseline can be determined by referring to the graph of the previous 10 min; where: (1) fetal tachycardia (tachycardia): refers to a fetal heart baseline >160 beats/min for ≥10 min; (2) fetal bradycardia (bradycardia): refers to a fetal heart baseline <110 beats/min for ≥10 min.

**FIGURE 1:**
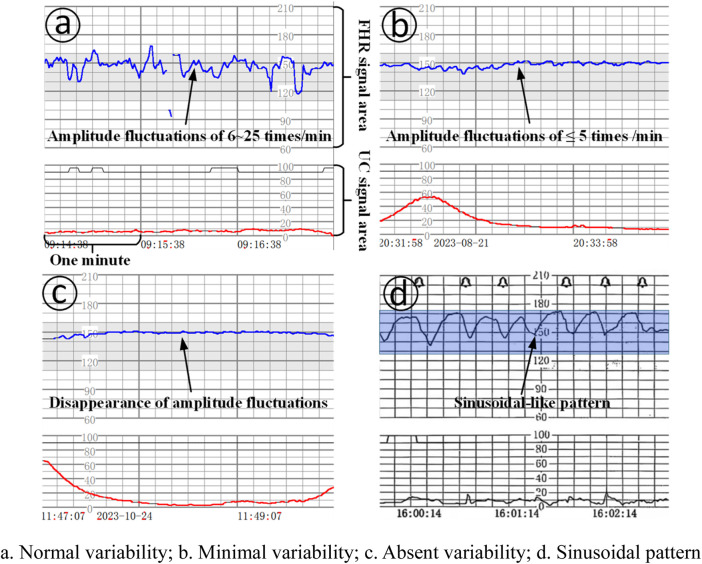
Baseline variation and sinusoidal pattern. **(a)**. Normal variability; **(b)**. Minimal variability; **(c)**. Absent variability; **(d)**. Sinusoidal pattern.

**FIGURE 2:**
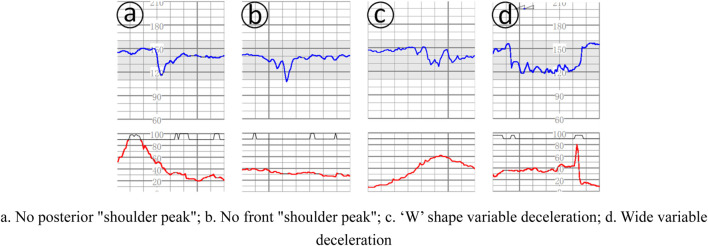
Several complex variable deceleration. **(a)**. No posterior “shoulder peak”; **(b)**. No front “shoulder peak”; **(c)**. ‘W’ shape variable deceleration; **(d)**. Wide variable deceleration.

**FIGURE 3:**
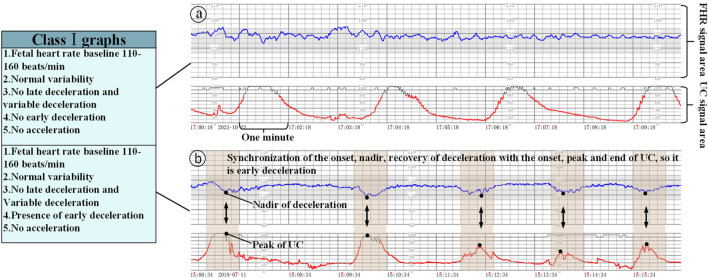
Class Ⅰ graphs (The x-axis represents the time and the y-axis is the corresponding FHR/UC signal). **(a)** Class I graphics with UC without acceleration/deceleration, **(b)** Class I graphics with UC and deceleration.

Baseline variability: refers to the change in amplitude of the fetal heart rate per minute from the peak to the trough, which can be visualized and quantified, of which: (1) absent variability: refers to the disappearance of amplitude fluctuations, as shown in Figure 1c; (2) minimal variability: refers to amplitude fluctuations of ≤5 times/min, as shown in Figure 1b; (3) normal/moderat evariability: refers to amplitude fluctuations of 6–25 times/min, as shown in Figure 1a.

Acceleration: refers to a sudden and significant increase in baseline fetal heart rate with a start-to-peak time of <30 s. The time from the start of the acceleration of the fetal heart rate to its return to the baseline fetal heart rate level is the time of acceleration. (1) Before 32 weeks of gestation, acceleration is ≥ 10 beats/min at the baseline level and lasts ≥10 s, but <2 min; (2) At 32 weeks of gestation and later, acceleration is ≥ 15 beats/min at the baseline level and lasts ≥15 s, but <2 min; (3) prolonged acceleration: it refers to an increase in the fetal heart rate that lasts ≥ 2 min, but <10 min; (4) if acceleration lasts ≥10 min, the baseline change in fetal heart rate is taken into consideration.

Deceleration: (1) early deceleration (ED): deceleration accompanied by contractions, usually symmetrical, slow decline to the nadir and then return to the baseline, the time from the beginning to the nadir ≥30 s, the nadir of deceleration is often coincident with the peak of contractions; in general, the beginning of deceleration, the nadir, the recovery Generally, the onset, nadir, and recovery of deceleration are synchronized with the onset, peak, and end of contractions; (2) late deceleration (LD): deceleration accompanied by contractions, usually symmetrically and slowly decreasing to the nadir and then recovering to baseline, with the onset to nadir time ≥30 s, and the nadir of deceleration is usually delayed from the peak of contractions. In general, the onset, nadir, and recovery of deceleration lag behind the onset, peak, and end of contractions, respectively; (3) Variable deceleration (VD): refers to a sudden, significant, and rapid decline in fetal heart rate, with an onset-to-nadir time of <30 s, a decline of ≥15 beats/min, and a duration of ≥15 s, but < 2 min. When varied deceleration is accompanied by contractions, the onset of deceleration is usually delayed by the peak of contractions. Deceleration is accompanied by contractions, and there is no fixed pattern between the onset, depth and duration of deceleration and contractions, as shown in Figure 2.

Uterine contraction: (1) normal uterine contraction (normal uterine activity): ≤5 times/10 min uterine contraction, observe for 30 min, and take the average; (2) uterine contraction is too frequent (tachysystole) (2) tachysystole: >5 contractions/10 min, 30 min of observation and take the average value.

Sinusoidal pattern: clearly visible, smooth, sinusoidal-like pattern, long variant of 3-5 cycles/min, lasting ≥20 min, and no acceleration exists, as shown in Figure 1d.

### 2.2 Interpretation and classification of CTG graphics during delivery

Class I graphs: The following conditions must be met: (1) the baseline fetal heart rate is 110–160 beats/min; (2) the baseline variation is normal variation; (3) there is no late deceleration and variant deceleration; (4) there is the presence or lack of early deceleration; and (5) there is the presence or lack of acceleration, which suggests that fetal acid-base balance is normal, as shown in Figures 3a,b.

**FIGURE 4:**
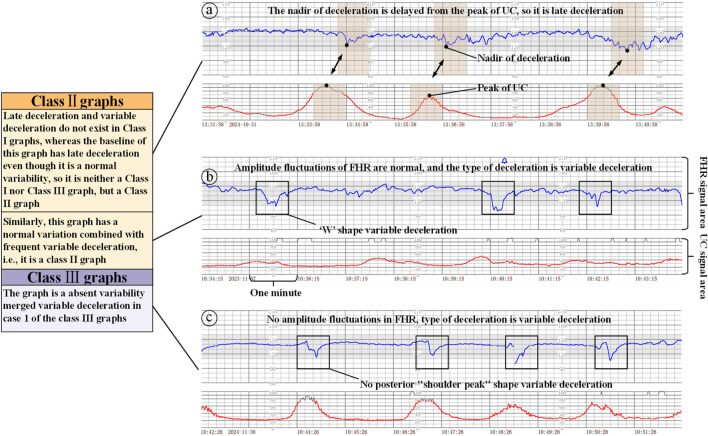
Class II/III graphs (The x-axis represents the time and the y-axis is the corresponding FHR/UC signal). **(a)** Class II graphics with UC and late deceleration, **(b)** Class II graphics with minimal variability, **(c)** Class III graphics with UC and absent variability.

Class II graphs: All cases other than Class I and Class III electronic fetal heart rate monitoring graphs are classified as Class II. It is not possible to interpret the presence of fetal acid-base balance disorders, but a combination of the clinical situation, continuous fetal heart rate monitoring, and other methods of assessment should be used to determine the presence or absence of fetal hypoxia, and intrauterine resuscitation may be required to improve the condition of the fetus, as shown in Figure 4a.

Class III graphs: (1) Fetal heart rate baseline absent variability and any of the following conditions are present: ① recurrent late decelerations; ② recurrent variable decelerations; ③ fetal bradycardia (fetal heart rate baseline <110 beats/min). (2) Sinusoidal pattern: It suggests that there is an acid-base balance imbalance in the fetus, i.e., fetal hypoxia, and appropriate measures should be taken immediately to correct the fetal hypoxia, including changing the position of the pregnant woman, administering oxygen, discontinuing the use of oxytocin, suppressing contractions, and correcting the hypotension of the pregnant woman, etc. If none of these measures work, the pregnancy should be terminated in an emergency, as shown in Figure 4.

### 2.3 The role of UC in fetal heart rate monitoring interpretation criteria

When interpreting class I graphs, it is necessary to combine with UC to determine whether there is ED or LD (Mendis et al., 2025), as well as the absent variability plus recurrent late deceleration in class III graphs, and failure to combine with contractions may lead to the misclassification of many graphs that should be classified as class I or class III as class II graphs. For example, we know that Figure 3b is a Class I graph and Figure 4a is a Class II graph, but it is difficult to differentiate between the two if we only look at the fetal heart rate without looking at that curve of contractions, and there is a possibility of misclassifying a Class I graph as a Class II graph and thus triggering unnecessary intervention, or on the contrary, misclassifying a Class II graph as a Class I graph and failing to intervene in a timely manner. Therefore, the temporal relationship between deceleration pattern and contraction is essential in the interpretation criteria of fetal cardiac monitoring charts. The FHR signal alone will increase the rate of misjudgment, resulting in a unimodal model that is prone to misjudging physiological fluctuations as pathological decelerations; for the two types of b and c in Figure 4, which only require a single signal from the FHR, can be identified, but for the pathological conditions of a, b in Figure 3, and a in Figure 4 and the absence of variability plus late decelerations in the class III graph, the pathological conditions cannot be accurately identified.

In terms of pathophysiological mechanisms, the synergistic changes of FHR and UC directly reflect the compensatory state of the fetal-placental unit, for example, the sudden decline of variant deceleration (VD) is associated with vagal reflexes due to cord compression, but its clinical significance needs to be combined with the timing of the occurrence of the out-of-contraction cycle to differentiate between episodic compression or persistent hypoxia, and the bimodal data can capture this dynamic interaction feature through time-domain alignment, whereas the single FHR signal provides only isolated information on heart rate fluctuations.

## 3 Methods

This chapter describes the datasets, network models, and attention mechanisms used in the experiment. First, the public dataset (Section 3.1) is summarized, the experimental data screening method (Section 3.2) is described, and then the network structure (Section 3.3) and its core attention mechanism (Section 3.4) are described in detail.

### 3.1 Publicly available dataset descriptions

The publicly available dataset CTU-CHB (published by the Czech Technical University and Brno University Hospital) is widely used as a baseline data source in the current field of fetal heart monitoring research. This dataset was created by screening 9,164 original fetal monitoring records collected during 2010–2012, and 552 CTG samples with complete clinical annotation were retained (Romagnoli et al., 2020). Although its data size and openness facilitate algorithm development, the following key shortcomings constrain its clinical value:

#### 3.1.1 Insufficient racial generalization

The CTU-CHB dataset contains data from only a single population of white European pregnant women, whose FHR and UC signaling characteristics show a high degree of homogeneity. However, the physiologic dynamics of the target clinical scenario (a group of Chinese pregnant women) may have geographic or population-specific patterns (e.g., baseline heart rate offset, differences in contraction pressure response, etc.). This data distribution bias leads to difficulties in generalizing models trained on a single population to heterogeneous populations, which in turn triggers cross-domain decision bias.

#### 3.1.2 Lack of multimodal data integrity

The vast majority of samples in the dataset had incomplete or missing UC signals, forcing studies using this dataset to analyze only a single FHR channel (Francis et al., 2024). This unimodal modeling approach is a serious deviation from the clinical guideline of “two-parameter synergistic assessment” (i.e., simultaneous monitoring of the time domain/frequency domain coupling of FHR and UC). For example, early signs of fetal hypoxia are often characterized by a rise in contraction pressure accompanied by an absence of accelerated fetal heart rate, and a single FHR signal cannot capture such dynamic correlations.

#### 3.1.3 Introduction of bias by data preprocessing methods

For the missing values of FHR signals, existing studies commonly use interpolation methods such as linear interpolation or spline interpolation to fill them (Li et al., 2025). Although such methods can achieve sequence continuity, their mathematical smoothing process destroys the original features of biological signals, and there is a certain degree of subjectivity in the filling of missing values of data, which results in the inability of the model to learn the real physiological response patterns.

### 3.2 Datasets used

**FIGURE 5:**
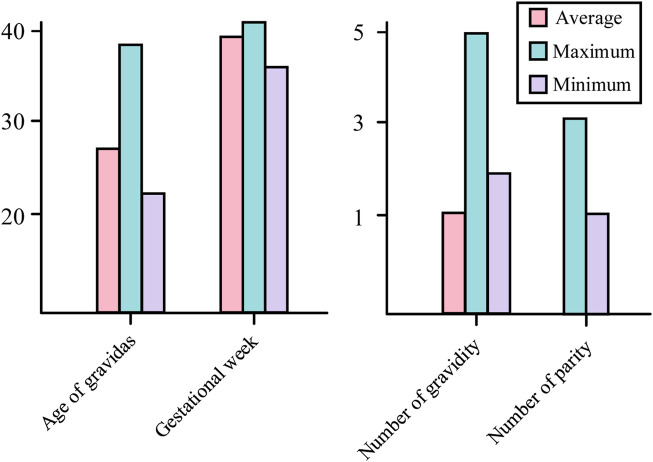
Information of pregnant women used in this study.

In order to break through the bottleneck of adaptability of public datasets in regional clinical applications, this study, in conjunction with the Obstetrics Center of Chengdu Tertiary Hospital, constructed a dedicated dataset for Chinese pregnant women. This dataset consecutively included 326 singleton pregnant women who underwent routine fetal heart rate monitoring in the obstetrics outpatient clinic of the First People’s Hospital of Longquanyi District, Chengdu City, Sichuan Province, China, from April 2019 to November 2023, with the information of the pregnant women as shown in Figure 5 those with severe fetal abnormalities, maternal arrhythmia, and signal loss >5 min were excluded. Independently labeled by two obstetricians with ≥5 years of experience according to FIGO 2015 guidelines; disagreements were arbitrated by a third person. Category distribution: 224 normal, 102 pathological. The study was approved by the Ethics Committee of the First People’s Hospital of Longquanyi District, Chengdu City, Sichuan Province, China (approval number: AF-KY-2024014), and all subjects signed informed consent.

Compared with the CTU-CHB dataset, its core advantages are reflected in three aspects: first, optimizing the signal acquisition parameters and evaluation thresholds for the unique physiological characteristics of Chinese pregnant women (e.g., the baseline mean fetal heart rate of 142 ± 8 bpm is significantly lower than that of 148 ± 10 bpm in the European population); second, realizing 100% synchronous acquisition of the FHR and UC signals with time alignment (sampling frequency of 4 Hz, time stamp error of Secondly, 100% synchronous acquisition and time alignment of FHR and UC signals (sampling frequency 4 Hz, time stamp error ≤0.25 s) was achieved to support Coupling Oscillation Analysis (COA), which meets the requirements of the clinical guidelines on the joint interpretation of multi-parameters; thirdly, data interpolation and filling techniques were strictly prohibited to maximally retain the original nonlinear characteristics of the biological signals. It provides infrastructure support for the subsequent multi-center validation and assessment of model generalization ability.

### 3.3 Dataset preprocessing

**FIGURE 6:**
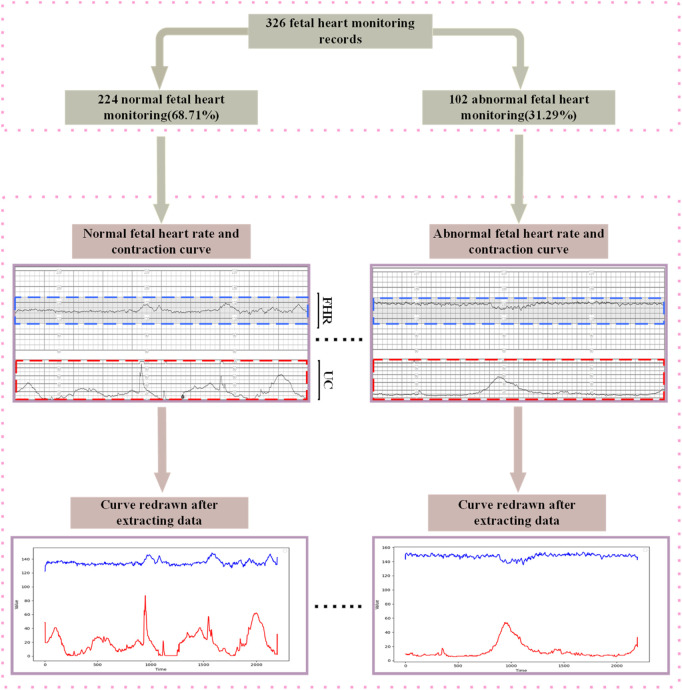
Flow chart of extracting FHR and UC signals from raw data.

Aiming at the grid shadow interference problem in CTGs collected from hospitals, this study proposes a noise suppression method based on image processing and template matching, and the complete flow is shown in Figure 6. The algorithm takes the original fetal monitor image set *I* = {*I*_1_,*I*_2_, … ,*I*_*N*_}∈ $R^{m\times n\times 3}$ (resolution m = 1,653, n = 2,339) as the input, and achieves the accurate extraction and standardization of the signal trajectory through the following steps:

Step 1: Image preprocessing and region segmentation: The color image *I*_*k*_ is first grayscaled by reading the guardianship recording image in grayscale format with a resolution of 1,653 × 2,339 pixels and binarized by setting a fixed threshold *τ* = 50 to convert the original image into a black and white binary image (Jia et al., 2023), where the fetal heart rate and contraction curve regions are labeled with foreground value of 1 and the background region of 0. The grayscale value *G*_*k*_ (*x*,*y*)is computed followedby an empirical thresholding for binarized segmentation Equations 1, 2 illustrate the computational process of its segmentation:

$$G_{k}\left(x,y\right)=\frac{1}{3}\sum_{c\in \left\{R,G,B\right\}}I_{k}\left(x,y,c\right)$$ (1)

$$B_{k}\left(x,y\right)=\left\{\begin{array}{l} 1\;{\;G}_{k}\left(x,y\right)>\tau \\ 0\;otherwise \end{array}\right.$$ (2)

where, $I_{k}\in R^{m\times n\times 3}$ denotes the original fetal monitor color image (the k-th), *G*_*k*_ (*x*,*y*)∈[0,255] denotes the pixel values after grayscaling, with coordinates of the x-th row and y-th column in the image, and *B*_*k*_(*x*,*y*)∈{0,1} denotes the binarized mask, which is used for segmenting the signal track region, and*τ* = 50 is the empirical threshold, which is an operation that can efficiently preserve the FHR and UC signal trajectory region, while filtering out the background grid interference.

Step 2: Physiological signal template modeling: two types of physiologic signal templates are defined based on the mapping relationship between clinical ranges and image scales:

1. Vertical scanning of the image in the region of rows 281 to 569, which corresponds to the band of the fetal heart rate curve in the paper record chart. The clinical range of 60–210 bpm was simulated by a preset linear template to match the binarized image column by column, and the weighted average of the valid signal points in each column was calculated, and the final output of the standardized fetal heart rate signal sequence, FHR template *T*_*FHR*_(*S*) the FHR signal template is shown in Equation 3.

$$\begin{array}{c} T_{FHR}\left(S\right)=k_{FHR}\cdot \left(\frac{y_{\max}-y_{\min}}{S_{end}-S_{start}}\left(S-S_{start}\right)+y_{\min}\right)\\ +C_{FHR} \end{array}$$ (3)

where *k*_*FHR*_ = −1 denotes signal reflection (image longitudinal coordinates are opposite to the physical range), *C*_*FHR*_ = *y*_max_ + *y*_min_ is a compensation constant used to align the baseline after inverse mapping to the image coordinate system,the pixel range of the scale region for FHR is *S*∈[*S*_*start*_, *S*_*end*_), and the corresponding clinical range is *S*∈[*S*_*start*_, *S*_*end*_).

2. Similarly, the contraction pressure curve bands in the region of rows 628 to 770 of the scanned image were combined with a linear template of 0–100 mmHg to extract the contraction signals for each column, UC template*T*_*UC*_(*S*) the UC signal template is shown in Equation 4.

$$T_{UC}\left(S\right)=k_{UC}\cdot \left(\frac{y_{\max}^{\prime }-y_{\min}^{\prime }}{S_{end}^{\prime }-S_{start}^{\prime }}\left(S-S_{start}^{\prime }\right)\right)+C_{UC}$$ (4)

where *k*_*UC*_ = −1 denotes signal reflection, *k*_*UC*_ = −1 ensures baseline zeroing, the pixel range of the scale region of the UC is *k*_*UC*_ = −1, and the corresponding clinical range is y $\in \left[y_{\min}^{\prime },y_{\max}^{\prime }\right]$ .

Step 3: Longitudinal Signal Extraction and Noise Suppression: For each time point i, the longitudinal column of pixel data is extracted vertically, the binary signal is multiplied with the physiological template, and the mean value is computed only for the valid data points, i.e., those with *B*_*k*_ = 1, to suppress the noise, which is given by the following the calculation process is shown in Equations 5, 6.

$$S_{1}^{\left(k\right)}\left(i\right)=\frac{1}{\left|\Phi_{1}\right|+\varepsilon }\sum_{s\in \Phi_{1}}B_{k}\left(s,i\right)\cdot T_{FHR}\left(S\right)$$ (5)

$$S_{2}^{\left(k\right)}\left(i\right)=\frac{1}{\left|\Phi_{2}\right|+\varepsilon }\sum_{s\in \Phi_{2}}B_{k}\left(s,i\right)\cdot T_{UC}\left(S\right)$$ (6)

where, *i*∈[0,n]denotes the timeline pixel position, $S_{p}^{\left(k\right)}\left(i\right)$ denotes the p-th class signal in the k-th image (p = 1: FHR, p = 2: UC), Φ_*p*_ denotes the set of valid pixels for the p-th class signal, and *ε* = 10^−6^ avoids division by zero error. This operation generates a normalized time series by suppressing the random noise in the non-track region.

Step 4: Time series matrix construction; Perform character area localization the constructed matrix is given by Equation 7.

$$X=\left[\begin{array}{cc} \begin{array}{c} S_{1}^{\left(1\right)}\\ \begin{array}{c} S_{1}^{\left(2\right)}\\ \vdots \end{array} \end{array} & \begin{array}{c} S_{2}^{\left(1\right)}\\ \begin{array}{c} S_{2}^{\left(2\right)}\\ \vdots \end{array} \end{array}\\ S_{1}^{\left(N\right)} & S_{2}^{\left(N\right)} \end{array}\right]\in R^{2N\times n}$$ (7)

where *X* denotes the two-channel time series matrix after signal extraction, *N* denotes the total number of images, 2*N* is the number of rows (each recorded image has two signal channels, FHR and UC), and n is the number of columns i.e., the length of the time series.

### 3.4 Network model

Compared with other domains, medical image data usually has a small data volume, so the models should be prioritized to lightweight type to fit the data missingness (Chen et al., 2024). Although many image processing models such as Vision Transformer (Khan et al., 2022) and Swin Transformer (Liu Z. et al., 2021) have achieved good results in recent years, they require large data volumes to support them. Obviously, the use of large models leads to their overfitting problems on small datasets and high computational resource requirements, which makes it difficult to be efficiently deployed in resource-constrained healthcare scenarios.

DenseNet121 (Huang et al., 2017) (Densely connected Convolutional Networks) is a deep convolutional neural network whose core idea is to enhance feature propagation by means of dense connections in a Dense Block. First, each layer in the Dense Block is connected to all previous layers, and given an input image*X*_0_, it is forward propagated through a convolutional neural network (DenseLayer) containing*L*layers. Each layer L (1≤ L ≤L) performs a nonlinear mapping $H_{L}\left(\cdot \right)$ , which consists of a combination of the basic blocks of batch normalization (BN), ReLU activation function, and convolutional operations. The feature output of the L -th layer is denoted as ${\;X}_{L}$ .

**FIGURE 7:**
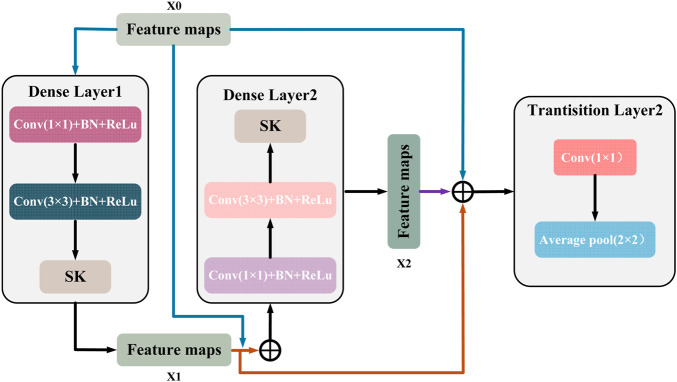
The internal structure of DenseBlock.

This connection makes the features fully reused and fused between different layers, enhances the feature transfer efficiency, and the gradient can be transferred more efficiently in the backpropagation process. Figure 7 shows a DenseBlock containing 2 DenseLayers, the L -th layer receives $X_{0},X_{1},\ldots \ldots X_{L-1\;}$ from all previous layers as input as shown in Equation 8:

$$X_{L}=H_{L}\left(X_{0},X_{1},\ldots \ldots ,X_{L-1}\right)$$ (8)

**FIGURE 8:**
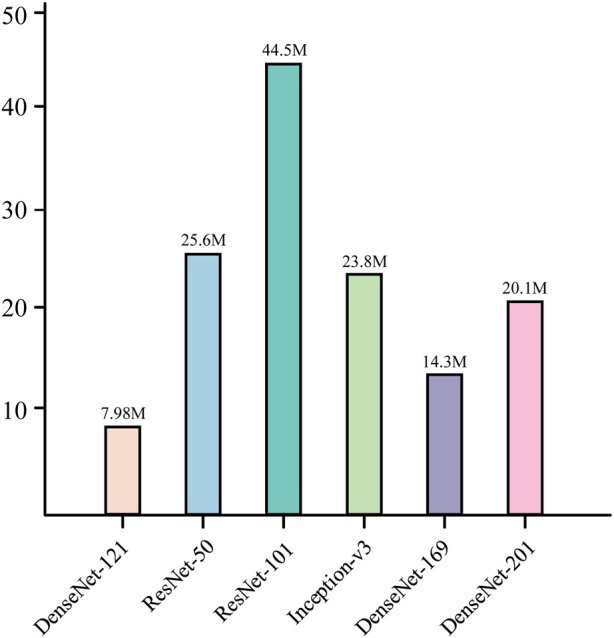
Parameter comparison of the model.

**FIGURE 9:**
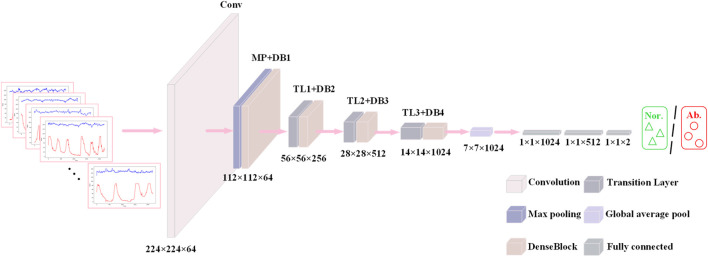
The DenseNet network structure.

This structural design allows the network to converge faster during the training process and reduces the occurrence of the gradient vanishing problem. At the same time, it is also characterized by high parameter efficiency; compared with other convolutional neural networks of the same type, DenseNet121 has fewer parameters at the same performance level (He et al., 2016; Szegedy et al., 2016), which is only 7.98M, as shown in Figure 8. In addition, there is a transition layer in the middle of every two Dense Block blocks, which contains 1 × 1 convolution with average pooling, actively reduces the feature map dimension through channel compression, and compression suppresses overfitting and enhances noise robustness. The DenseNet121 network structure is shown in Figure 9.

In the fetal heart map classification task, DenseNet121 fuses the shallow features of the signal with the deeper features through dense connections, enabling the network to extract rich feature information from different levels. This dense connectivity structure enables the network to better capture subtle features in fetal heart maps, such as short-term details: instantaneous fluctuations in fetal heart rate (e.g., variable deceleration) or impulse noise in contraction signals (maternal motion interference) (Chen et al., 2025), and long-time trends: contraction cycles (10–15 min) with baseline variability in fetal heart rate,

DenseNet121 progressively fuses features at different scales through 3 × 3 convolution cascaded in multiple layers within a dense block without relying on complex data enhancement or pre-training strategies, a feature that is crucial for capturing the synchronization of contraction peaks with fetal heart rate deceleration (Deceleration-Contraction Coupling).

This feature fusion approach not only improves the representativeness of the features, but also allows the network to better adapt to the complexity of fetal heart maps and improve the classification accuracy.

In the fetal heart map classification task, this means that DenseNet121 is able to achieve higher classification accuracy without increasing the computational burden. This is especially important for practical clinical applications, as its lightweight design not only reduces the computational resource requirements, but also improves the generalization ability of the model, making it ideal for scenarios with limited fetal heart image data.

### 3.5 Attention

Although the dense connectivity of DenseNet can aggregate multi-scale features (e.g., transient fluctuation and baseline drift) across layers, the fixed receptive field of its convolutional kernel makes it difficult to dynamically adapt to pathological patterns with different spatiotemporal characteristics. Fetal heart deceleration during the UC Peak Phase requires a large receptive field to capture cyclic correlations, while Beat-to-beat Variability relies on local detail extraction, but the fixed size of conventional convolution kernels limits the model’s ability to capture multi-scale physiological dynamics (Li et al., 2023; Zhang et al., 2023), and the noise of the fetal cardiogram is not consistent with the physiological events (deceleration) interference differs significantly from key physiological events (delayed deceleration) in the channel dimension, but traditional dense connectivity assigns equal weight to all feature channels, resulting in insufficient sensitivity of the model to low signal-to-noise ratio regions.

**FIGURE 10:**
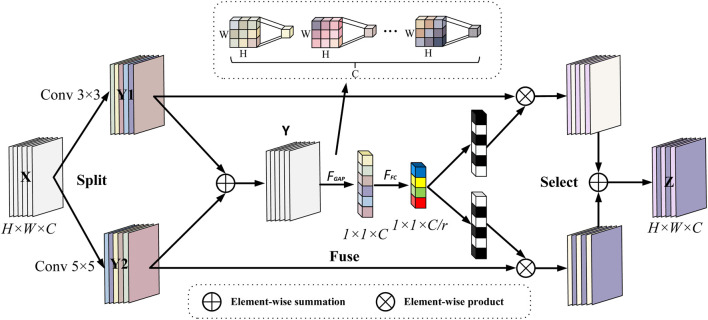
Selective kernel convolution.

To address the problem of limited data volume and complex pathology features in fetal heart maps, this study further introduces the Selective Kernel (SK) (Li et al., 2019a) attention module, which is inserted after each DenseLayer of the DenseBlock. The dense connectivity of DenseNet121 provides an ideal architectural foundation for this purpose. The multi-scale feature maps (abstraction layers of different Dense Blocks) output by the dense connectivity provide rich inputs for the dynamic sense field selection of the SK module, which enhances the feature response to key phases of contractions (e.g., peak periods) through Channel Recalibration, while the dense connectivity ensures that local details (e.g., small fluctuations in fetal heart rate) are not forgotten by the deep network forgotten by the deep network. SK convolution is implemented by three operations, Split, Fuse and Select, the process of which is depicted in Figure 10.

*Split*: two convolution kernels of sizes 3 and 5 are used to perform convolution operations on the input features (each convolution operation is a set of GBRs), i.e., $F_{1}:X\to Y_{1}\in R^{H\times W\times C}$ and $F_{2}:X\to Y_{2}\in R^{H\times W\times C}$ to obtain two-scale feature representations and, for efficiency, a 3 × 3 convolution kernel with a null size of 2 is used instead of the 5 × 5 conventional convolution kernel. The same input is fed into two “stethoscopes” simultaneously: 3 × 3 convolution → captures the instantaneous variation (beat-to-beat) over 0.2–0.4 s; 5 × 5 (null = 2) convolution → covers the contraction cycle over 0.8–1.2 s correlation. The two branch outputs *Y*_1_ and *Y*_2_were identical in shape, facilitating subsequent pixel-by-pixel fusion.

*Fuse*: in order to enable neurons to adaptively adjust the size of their receptive fields according to the content of the stimulus, the results of the two branches are first fused by elemental summation, i.e., the corresponding elements in a tensor of the same shape are summed:

$$Y=Y_{1}+Y_{2}$$ (9)

then the global information is then embedded through global average pooling to generate the channel statistics for s ∈ R^C, specifically, the cth element of s computed through the spatial dimensions *H* × *W* shrinking *Y*.

$$s_{c}=F_{GAP}\left(Y_{c}\right)=\frac{1}{H\times W}\sum_{i=1}^{H}\sum_{j=1}^{W}Y_{c}\left(i,j\right)$$ (10)

this step is equivalent to an obstetrician quickly going through the entire curve and noting which bands are abnormal in energy.

Then, a fully connected operation is performed on the channel statistic S containing global information to obtain the low-dimensional eigenvector *z* after dimensionality reduction and abstraction, which retains the key information of the input and significantly reduces the dimension, so as to reduce the parameters of the subsequent attention layer and improve the inference speed:

$$z=F_{FC}\left(s\right)=\delta \left(B\left(Ws\right)\right)$$ (11)

where $W\in R^{d\times C}$ , B denotes batch normalization, *δ*is the ReLU function, and z has dimension *d*. The formula is as follows as shown in Equation 12:

$$d=\max\left(\frac{C}{r},L\right)$$ (12)

where r is the ratio of dimensionality reduction, when r is larger sacrifice part of the expression ability to improve efficiency, suitable for small models/lightweighting, so in this study, *r* is set to 16 and *L* = 32 is the lower limit value, in order to prevent over-compression of the information, to ensure that the ability of expression.

*Select*: the input is the feature compact descriptor *z*, through the cross-channel soft attention mechanism, that is, through the attention weight, the information that dynamically determines which branch each channel should focus on, through exponential operation and normalization, the score is converted into a probability value *a*_*c*_and *b*_*c*_, satisfying *a*_*c*_ + *b*_*c*_ = 1, and its mathematical expression is:

$$a_{c}=\frac{e^{A_{c}z}}{e^{A_{c}z}+e^{B_{c}z}},b_{c}=\frac{e^{B_{c}z}}{e^{A_{c}z}+e^{B_{c}z}}$$ (13)

where $A,B\in R^{d\times c}$ represent the learnable parameter matrix, and the learnable matrix *A*,*B* maps *z* into two probabilities *a* and *b*. *a*≈1, *b* ≈ 0: the model believes that the current channel should be dominated by instantaneous details, such as at the starting point of mutation deceleration; *a*≈0, *b* ≈ 1: the model pays more attention to long-term trends, such as determining whether the baseline continues to decline at peak contractions; a and b between 0 and 1: the model blends the two scales to adapt to the transition interval. Each row of each matrix corresponds to a channel of weight calculation, with a, b representing the *Y*_1_ and *Y*_2_ soft attention vectors. Note: $A_{c}{,B}_{c}\in R^{1\times d}$ represents the c row of the matrix *A*,*B* corresponding to the weight parameter of the c channel, and *a*_*c*_, *b*_*c*_ represents the c element of *a*,*b*. Finally, feature fusion:

$$V_{c}=a_{c}\cdot Y_{1c}+b_{c}\cdot Y_{2c}$$ (14)

where *V* = [*V*_1_,*V*_2_, … … ,*V*_*c*_], $V_{c}\in R^{H\times W}$ , the final output *V*_*c*_ is equivalent to adjusting the volume of the two stethoscopes in real time according to “clinical importance”.

The Selective Kernel (SK) attention mechanism used in this study achieves intelligent focusing on key pathological features in fetal heart-contraction bimodal signals through dynamic gating weights, and its core innovation is to mimic the process of diagnostic cognition of irregular physiological events by obstetricians. During contraction stress, the SK module generates feature mappings of differentiated receptive fields through parallel processing of multibranch convolutional kernels (Equation 9) - 3 × 3 kernels capture transient variability (e.g., subtle fluctuations in beat-to-beat variability), whereas null convolution of equivalent 5 × 5 kernels captures cyclic associations (e.g., lag phase difference between deceleration and contraction). Global average pooling (Equation 10) compresses the spatiotemporal features into a channel statistic s, which essentially quantifies the energy distribution of different frequency components. The fully connected layer (Equation 11) constructs in effect a low-dimensional streaming representation of the dynamic properties of the signal by means of an abstract feature vector z extracted from the bottleneck structure (*r* = 16), where each dimension corresponds to a typical pathological pattern.

The calculation of gating weights (Equation 13) realizes the embedding of clinical *a priori* knowledge through the learnable parameter matrix *A*,*B*- when the input signal has a contraction-triggered steep drop (e.g., a W-type valley of variability deceleration), the weights of the larger receptive field branches (5 × 5 equivalent kernels) *a*_*c*_ are automatically augmented by the *Softmax* function (>0.7), allowing the model to prioritize the temporal relationship between the overall pattern of deceleration and the contraction cycle; conversely, when subtle fluctuations are detected (e.g., baseline variability decay), the weights *b*_*c*_ of the smaller receptive field branches (3 × 3 kernels) are elevated, focusing on local slope changes. This adaptive selection mechanism (Equation 14) achieves a triple optimization at the physiological level: 1) in the time domain, the dynamic weight assignment strengthens the characteristic response of the critical phase of contraction (15 s after the peak), and weakens the redundant information of the inter-contraction interval; 2) in the frequency domain, the interference of the maternal motion artifacts (high-frequency noise) is suppressed by the channel re-calibration, and the hypoxia-associated fluctuation in the frequency band of 0.04–0.15 Hz is enhanced; 3) Spatially, multi-scale feature fusion ensures that transient but clinically significant signal transitions (e.g., W-shaped double valleys of variable deceleration) are not smoothed by the fixed receptive fields of conventional convolution, and automatically enhances the detection sensitivity of subtle but prognostically critical signal turning points (e.g., deceleration recovery slopes <1 bpm/s) during the contraction stress phase to maximally mimic the obstetrician’s interpretation process.

## 4 Experimental results and discussion

### 4.1 Experimental setup

This study is based on PyTorch 2.6.0 and CUDA 12.0 as a deep learning framework to build neural network models, the ratio of the training set to the test set is 7:3, and the experiments are all run on the NVIDIA RTX4060 equipped with AMD 7735, and 16 GB DDR5, and the hyperparameters are shown in Table 1.

**TABLE 1:** Hyperparameters for proposed method.

Hyperparameter	Value
K	32
Epochs	100
Learning rate	0.001
Batch Size	32
Loss Function	FocalLoss
Optimisation	Adam

### 4.2 Evaluation indicators

Because of the uneven proportion of data, in order to more fully validate the performance of the model in this study, several metrics such as precision, recall, F1 score, confusion matrix and subjects’ work characteristic curves (ROCs) and area under the ROC curve (AUCs) were introduced for assessment.

In this assessment framework, CTG plot normal is defined as positive category and abnormal as negative category. Based on this setting, the model prediction results were defined as follows: cases in which the model correctly predicted fetal normality were called True Positive (TP); cases in which the model incorrectly predicted fetal abnormality as a positive category were called False Positive (FP); cases in which the model correctly predicted fetal abnormality as a negative category were called True Negative (TN); and the situation where the model incorrectly predicts fetal normal as a negative category is referred to as False Negative (FN).

The precision rate indicates the proportion of samples predicted to be in the normal/abnormal category that are actually in the normal/abnormal category and measures the ability of the model to avoid misdiagnosis the calculation method for precision is shown in Equations 15, 16.

$${Precision}_{Normal}=\frac{TP}{TP+FP}$$ (15)

$${Precision}_{Abnormal}=\frac{TN}{TN+FN}$$ (16)

Recall represents the proportion of true normal/abnormal samples correctly identified by the model to the total number of actual normal/abnormal samples, which reflects the model’s ability to capture normal/abnormal categories. In the fetal heart rate monitoring scenario, this metric is of key clinical significance: false positives will lead to missed high-risk cases and delayed necessary interventions (emergency cesarean section), thus jeopardizing the safety of mother and baby, while false negatives, although they may lead to over-medical interventions, have a significantly lower risk of adverse clinical outcomes than false-positive scenarios. Therefore, minimizing the proportion of FP by optimizing the recall rate is a central goal to guarantee the safety of decision-making in high-risk pregnancies and is highly consistent with the guideline of clinical priority to reduce the rate of missed diagnoses the recall rate is calculated as shown in Equations 17, 18.

$${Recall}_{Normal}=\frac{TP}{TP+FN}$$ (17)

$${Recall}_{Abnormal}=\frac{TN}{TN+FP}$$ (18)

The F1 score represents the reconciled mean of precision and recall and is used to balance the two the calculation method for F1 scores is shown in Equation 19.

$$F1-score=2\times \frac{Precision\times Recall}{Precision+Recall}$$ (19)

Accuracy indicates the number of correctly predicted samples as a proportion of the total number of samples the calculation method for accuracy is shown in Equation 20.

$$Accuracy=\frac{TP+TN}{TP+TN+FP+FN}$$ (20)

Confusion Matrix is a matrix structure for evaluating the performance of classification models (Valero-Carreras et al., 2023), which quantitatively presents the accuracy and error distribution of classification results by cross-referencing the true categories of the samples with the predicted categories of the model the confusion matrix is shown in Equation 21.

$$Confusion\;Matrix=\left[\begin{array}{cc} TP & FN\\ FP & TN \end{array}\right]$$ (21)

ROC is a visualization tool for evaluating the performance of a binary classification model, with the False Positive Rate (FPR) on the horizontal axis and the True Positive Rate (TPR) on the vertical axis. The ROC depicts the model’s ability to discriminate between positive and negative categories by traversing all the classification thresholds: the closer the curve is to the upper left corner (FPR approaches 0, TPR approaches 1), the better the classification performance, and the AUC is the area enclosed by the ROC curve and the coordinate axis to quantify the overall classification effectiveness of the model. The ROC curve depicts the model’s ability to discriminate between positive and negative categories by traversing all classification thresholds: the closer the curve is to the upper left corner (FPR tends to 0, TPR tends to 1), the better the model’s classification performance is, and the AUC is the area bounded by the ROC curve and the axes, which is used to quantify the model’s overall classification effectiveness. “When AUC = 0.5, the model is equivalent to a random guess; when AUC = 1, the model has the ability to classify perfectly, and its formula is the calculation method for AUC is shown in Equation 22.

$$AUC=\int_{0}^{1}ROC\left(t\right)dt$$ (22)

### 4.3 Results

**FIGURE 11:**
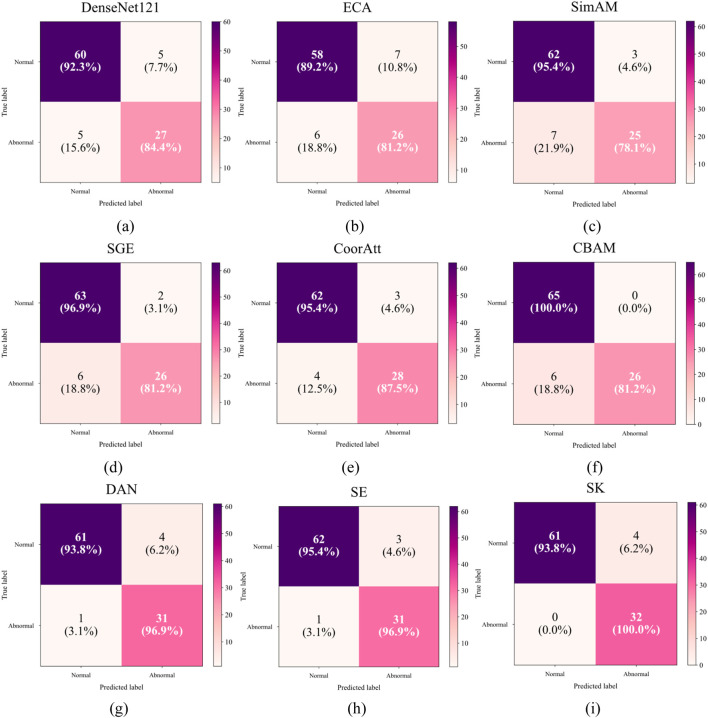
Confusion matrix for different attentions: **(a)** Only DenseNet121 **(b)** ECA **(c)** SimAM **(d)** SGE **(e)** CoorAtt **(f)** CBAM **(g)** DAN **(h)** SE **(i)** SK.

Table 2 compares the side-by-side comparisons using the DenseNet121 backbone network and fusion of eight mainstream attention mechanisms, in which SK achieves the optimal performance with an accuracy rate of 0.9588, which is tied for first place with classical SE attention, but demonstrates significant advantages in key clinical metrics: the normal samples achieve a 100% precision rate (Precision = 1.00), which effectively avoids the risk of misdiagnosing the normal The normal samples achieved 100% precision (Precision = 1.00), effectively avoiding the risk of misdiagnosing normal fetal heart as abnormal; the abnormal samples achieved 100% recall (Recall = 1.00), ensuring that all abnormal cases were effectively detected. In terms of the comprehensive assessment indexes, both the normal category F1-score (0.97) and the abnormal category F1-score (1.00) were significantly better than the comparison scheme, with an improvement of 5.0% and 16.0%, respectively, compared with the baseline method, and the confusion matrices are shown in Figure 11. The experimental results show that the SK module enhances the model’s hierarchical characterization of fetal heart fluctuation features through the strategy of dynamically selecting multi-scale convolutional kernels, and its channel attention mechanism precisely focuses on the pathology-related features, which results in a clearer decision boundary for the normal/abnormal category. This performance advantage is valuable in clinical scenarios to eliminate the waste of medical resources caused by false-positive diagnosis and avoid the medical risks caused by false-negative missed diagnosis.

**TABLE 2:** Comparison between using DenseNet alone and integrating other attention.

Attention	Accuracy	Normal			Abnormal		
		Precision	Recall	F1-score	Precision	Recall	F1-score
DenseNet121 (Huang et al., 2017)	0.8969	0.92	0.92	0.92	0.84	0.84	0.84
ECA (Wang et al., 2020)	0.8660	0.91	0.89	0.90	0.79	0.81	0.80
SimAM (Yang et al., 2021)	0.8969	0.90	0.95	0.93	0.89	0.78	0.83
SGE (Li et al., 2019b)	0.9175	0.91	0.97	0.94	0.93	0.81	0.87
CoorAtt (Hou et al., 2021)	0.9278	0.94	0.95	0.95	0.90	0.88	0.89
CBAM (Woo et al., 2018)	0.9381	0.92	1.00	0.96	1.00	0.81	0.90
DAN (Fu et al., 2019)	0.9485	0.98	0.94	0.96	0.89	0.97	0.93
SE (Hu et al., 2018)	0.9588	0.98	0.95	0.97	0.91	0.97	0.94
SK (Li et al., 2019a)	0.9588	1.00	0.94	0.97	0.89	1.00	0.94

Table 3 summarize the results of the evaluation of existing methods for fetal heartbeat monitoring classification, covering the performance of different models in machine learning, deep learning on their respective datasets. It can be seen that this study achieved AUC: 94.4/Acc: 95.88/F1: 97 on the self-constructed bimodal dataset, whereas the performance of unimodal (FHR only) dropped to AUC 81.2/Acc 87.69/F1 0.79 under the same model structure, a result that validates the value of contraction signals as an auxiliary feature. The comparison results in Table 3 do not constitute a strict performance ranking, and there are limitations in directly comparing the performance of these methods due to the following reasons: first, the cited studies each used a different private or public dataset, with sample sizes ranging from 83 to 4,473 cases, and the difference in sample sizes may affect the assessment of the model’s generalization ability. Second, some of the methods were designed based on 1D fetal heart rate signals, whereas our DenseNet121-SK model deals with 2D images after bimodal signal conversion. Even if the reproduction on the same dataset is forced, the signal needs to be resampled, windowed, or spectrally transformed, which introduces additional preprocessing bias and leads to less rigorous performance comparisons. Therefore, the comparison results in Table 3 are more of a reference for method trends rather than a strict performance ranking.

**TABLE 3:** Aggregate of existing methods and proposed methods use only the effect of monomodality.

Reference	Method	Evaluating indicator			Dataset used	Sample size	Performance (%)
		AUC	Acc	F1			
Krupa et al. (2011)	SVM		√		Private	90	87
Spilka et al. (2014)	NB,SVM,DT			√	Private	217	71.5
Czabanski et al. (2012)	WFS + LS-SVM		√		Private	186	92.0
Fanelli et al. (2013)	ST	√			Private	122	75
Dash et al. (2014)	GM,NB			√	Private	83	69.9
Stylios et al. (2016)	LS-SVM	√			CTU-UHB	552	72.81
Georgoulas et al. (2017)	LS-SVM	√			CTU-UHB	552	68.54
Cömert et al. (2018)	LS-SVM	√			CTU-UHB	552	64.64
Li et al. (2018)	CNN		√		Private	4,473	93.24
O’sullivan et al. (2021)	ARMA + SVM	√			CTU-UHB	552	86
Lin et al. (2024)	LARA	√			Private	114	87.2
Ours (Only FHR)	DenseNet121+SK	√	√	√	Private	326	81.2/87.69/79
Ours (FHR + UC)	DenseNet121+SK	√	√	√	Private	326	94.4/95.88/97

### 4.4 Disscussion

The following limitations of this study need to be accounted for: first, the limitations of the dataset size and geographic origin (single center in Southwest China) may lead to the model’s insufficient ability to generalize to specific populations (e.g., obese pregnant women); second, although the SK Attention module significantly improves the model’s performance (3.2% improvement in accuracy), its computational complexity is increased by approximately 15% compared to the base DenseNet121 (Liu et al., 2023; Tang et al., 2023), and the Optimization measures such as quantization compression may be required in extreme resource-constrained environments; third, due to the lack of publicly available bimodal fetal heart monitoring benchmark datasets, existing comparison experiments can only be compared with unimodal methods and traditional machine learning baselines, and this benchmark discrepancy may affect the objectivity of the performance evaluation; lastly, there is a lack of standardized signal preprocessing and annotation specifications in the current field of fetal heart monitoring, which makes it difficult to directly compare the results of different studies with each other. Results are difficult to compare directly. These limitations suggest the need for further research in multi-center large sample validation, computational efficiency optimization, and standardized baseline establishment.

## 5 Future plans

### 5.1 Multi-center retrospective validation

To assess the generalization ability of the model, this study plans to conduct multi-center validation jointly with several tertiary hospitals in the future. Each center independently collected 150 fetal cardiac monitoring samples (including 10% extreme pathology cases) to ensure no overlap with the training set. The following metrics were used to quantify model performance decay the calculation equation for GDR is shown in Equation 23.

$$GDR=\frac{{AUC}_{Training\;set}-{AUC}_{External\;set}}{{AUC}_{Training\;set}}\times 100\%$$ (23)

where GDR (Generalization Decay Rate) reflects the model cross-center performance decay rate. When GDR >15%, a domain adaptation fine-tuning strategy based on Maximum Mean Discrepancy (MMD) is initiated: the Adam optimizer (learning rate 1 × 10^−4^, batch size 16) is used to minimize the feature distribution of the source domain and the target domain in the RKHS space difference.

### 5.2 Special testing for high-risk groups

In order to verify the applicability of the model in high-risk pregnancy populations, special validation is planned to be carried out in the future for three high-risk subpopulations: gestational diabetes, preeclampsia and fetal growth restriction. The stratified sampling strategy is used to ensure that the samples of each subset are representative, and the ability of the model to identify pathologically-specific patterns (such as loss of acceleration in the gestational diabetes group and variation deceleration in the preeclampsia group) is emphasized. When the F1 value of a specific subset is verified to be less than 0.80, the Focal Loss function (γ = 2) will be used to retrain the subset samples to alleviate the problem of class imbalance. At the same time, an adversarial discriminator (gradient penalty coefficient λ = 0.3) was introduced to minimize the distribution differences between the source domain and the target domain in the feature space, and improve the generalization ability of the model to the characteristics of high-risk groups.

### 5.3 Future research directions

In the future, we will also study image segmentation models in the field of fetal heart rate monitoring, in order to use image segmentation technology to identify important parts such as acceleration, deceleration, and baseline in the fetal heart rate monitoring chart, as well as the most popular large language model in the field of AI recently.

## 6 Conclusion

**FIGURE 12:**
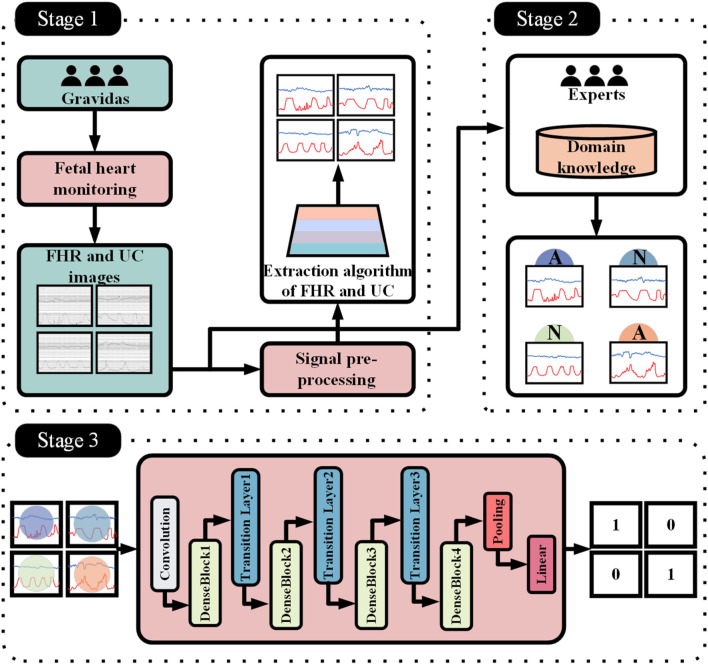
Flow chart of using the proposed method to assist diagnosis.

The model proposed in this study uses a bimodal (FHR + UC) signal fusion design to simulate the clinical habits of the two dynamically related clinical habits (such as the timing coupling of peak contractions and fetal heart rate deceleration), and its input form (including hyperbolic images) is highly consistent with the clinical interpretation scenario. It provides obstetricians with reliable objective opinions during CTG monitoring during childbirth and reduces misinterpretation caused by subjective differences. The introduction of the SK attention module dynamically adjusts the receptive field: small-scale convolution captures details for transient fetal heart rate fluctuations (e.g., beat-to-beat variations) and large-scale convolutional trends for contraction cycle associations (e.g., the lag relationship between late deceleration and contractions), which is consistent with the clinical focus on different pathological patterns and compensates for the shortcomings of DenseNet121s fixed receptive fields. The DenseNet121-SK architecture is only 8.3M (7.98M for the base DenseNet121 and 0.32M for the SK module), which guarantees 95.88% accuracy and 100% abnormal sample recall while low computational cost, allowing it to run on mid-range GPUs or high-performance CPUs without relying on high-end computing clusters. For primary medical institutions lacking GPUs, model quantization (such as INT8 precision) can further compress the computational requirements while reducing memory footprint, and the dataset is not filled with interpolation, retaining the original signal characteristics, reducing the dependence on complex preprocessing processes, and facilitating reuse in scenarios with simple data acquisition conditions (such as the original image output by fetal heart rate monitors in primary hospitals). These characteristics enable the system to effectively assist in clinical decision-making and provide a reliable basis for timely intervention in high-risk pregnancies, and Figure 12 shows the process of the proposed model assisting in the diagnosis of intrapartum CTG maps. However, it should be emphasized that the system output should always be used in conjunction with clinical evaluation to form a complete diagnosis and treatment decision-making chain.

## Data Availability

The data analyzed in this study is subject to the following licenses/restrictions: The data involves patient privacy, and a data confidentiality agreement has been signed with the hospital. Requests to access these datasets should be directed to Tianxin Qiu, 307397327@qq.com.
